# Ten years of *Genome Medicine*

**DOI:** 10.1186/s13073-019-0618-x

**Published:** 2019-02-15

**Authors:** Charles Auffray, Julian L. Griffin, Muin J. Khoury, James R. Lupski, Matthias Schwab

**Affiliations:** 1 European Institute for Systems Biology and Medicine (EISBM), Vourles, France; 2 Department of Biochemistry and Cambridge Systems Biology Centre, University of Cambridge, Sanger Building, Tennis Court Road, Cambridge, CB2 1GA UK; 3 Computational and Systems Medicine, Department of Surgery and Oncology, Imperial College London, London, SW7 2AZ UK; 4 Office of Public Health Genomics, Centers for Disease Control and Prevention, Atlanta, GA 30329 USA; 5 Department of Molecular and Human Genetics, Baylor College of Medicine, Baylor Plaza, Houston, TX 77030 USA; 6 Dr Margarete Fischer-Bosch Institute of Clinical Pharmacology, Auerbachstraße, 70376 Stuttgart, Germany; 7 Department of Clinical Pharmacology, University Hospital Tübingen, Auf der Morgenstelle, 72076 Tübingen, Germany

This year marks the 10th anniversary of *Genome Medicine*. The journal was launched to meet the need in the community for a platform to publish impactful and open science that advances basic and clinical research—using genetic, genomic, omic, and systems approaches—that has the potential to revolutionize the practice of medicine. We have seen the journal evolve along with the changing landscape of health and disease, including the increasing use of genome-scale approaches in medical research and clinical practice, the generation and analysis of patient- and population-level data, and the clinical implementation of these approaches in precision medicine and public health. *Genome Medicine*, guided by our renowned Section Editors, continues to serve an ever-growing community of interdisciplinary researchers. Here, our Section Editors discuss the major advances in the field and their applications in genomic medicine during the past decade.

## A decade of medical genomics

Major breakthroughs in medical genomics have been enabled by the implementation of clinical exome sequencing (ES) for molecular diagnosis, by genomics approaches for understanding the genetic basis of complex traits and diseases, and by progress in understanding genotype–phenotype relationships. Initially, two papers reported clinical ES at scale, describing ES results from 814 and 3386 consecutive patients with undiagnosed suspected genetic conditions; in both studies, the molecular diagnostic yield was around 25% [1, 2]. In the study by Yang et al. [2], an in-depth analysis of the first 2000 cases revealed that approximately 30% of patients with suspected genetic disease harbored presumptive causative mutations in disease genes that were discovered in the previous 3 years. These data portended that the rapid pace of disease gene discovery using genomics approaches would enable increasing molecular diagnostic rates with systematic reanalysis of clinical ES data [3–5].

Genomics approaches to congenital anomalies, such as the complex trait of congenital scoliosis (CS), have revealed a new genetic model for developmental birth defects whereby a rare variant null allele is combined with a non-coding hypomorphic variant allele to cause the disease phenotype [6]. The compound inheritance gene dosage model shows that haploinsufficiency does not cause the phenotype whereas a homozygous null allele is lethal. Developmental expression of less than 50% affects somitogenesis and results in the formation of hemivertebrae, causing CS. These findings show that both allelic heterogeneity and different combinations of alleles at a locus can be important for the manifestations of disease traits, that the combination of rare variant coding alleles with common variant non-coding alleles can be important for trait manifestation or for the penetrance of disease, and that gene dosage and expression perturbations can result in developmental birth defects.

Cognitive phenotypes such as developmental delay/intellectual disability (DD/ID), autism, and schizophrenia are complex traits. Nevertheless, the genetic underpinnings of these phenotypes have begun to be elucidated through genome-wide studies of copy number variants (CNV) and de novo mutations. The association of CNVs with cognitive phenotypes has been almost exclusively evaluated using clinically ascertained cohorts. By studying an unselected sample of approximately 8000 individuals in the Estonian BioBank, however, investigators identified known pathogenic CNVs in adult individuals in the general population that may be associated with unrecognized clinical sequelae [7]. Moreover, individually rare but collectively common intermediate-sized CNVs were shown to be associated negatively with educational attainment, implicating genetic variants in cognitive traits [7]. This study points to the need for better recognition and clinical phenotyping of perturbations of biological homeostasis in cognition to enable the diagnosis of DD/ID phenotypes and the further sub-categorization of cognitive disease traits.

Clinical ES can provide insight into the relationship between the observed clinical phenotypes and underlying genotypes. Two or more disease loci were involved in approximately 1 in every 20 patients among the 7374 patients for whom a putative molecular diagnosis was achieved in the study by Posey et al. [8]. Intriguingly, both variant alleles were de novo in 44.7% of patients who had two mono-allelic pathogenic variants. Interestingly, phenotypic similarity scores were significantly lower among patients in whom the observed clinical phenotype resulted from two distinct Mendelian disorders that affected different organ systems than in patients with disorders that had overlapping phenotypic features. Importantly, structured clinical ontologies, described using the Human Phenotype Ontology (HPO) terms, could be used to quantify the degree of overlap between two Mendelian diseases in the same patient [8]. Future clinical ES studies may capitalize further on the rich source of rare variant genomic data and on human phenotyping of the perturbations of biological homeostasis that present as disease.

*James R. Lupski*

*Section Editor, Genomics and epigenomics of disease*

## Public health genomics: where to next?

This year marks the 10-year anniversary of *Genome Medicine*, whereas 2018 marked the 20-year anniversary of the launch of public health genomics globally [9]. A lot has happened in the past two decades. Three issues have emerged at the forefront of applications of genomics in public health and these issues will continue to propel the field in the next few years.

First, we see an acceleration of the implementation of evidence-based genomic applications in medicine and public health. Most applications are still not ready for routine clinical practice, but a growing number are supported by evidence of clinical validity and utility sufficient to merit integration into practice. The Centers for Disease Control and Prevention (CDC) classifies and updates genomic applications at three levels of evidence [10], which can be found in the Public Health Genomics Knowledge Base of the CDC [11]. In addition to newborn screening, most applications are in the fields of cancer, pharmacogenomics, congenital disorders, and cardiovascular disease. Our collective challenge today is to use principles of implementation science to maximize population health benefits [11].

Second, model processes are emerging that allow whole genome or exome sequencing data to be evaluated and integrated within health systems as part of routine care, including the integration of sequencing data with electronic health records. Several health systems in the US and elsewhere are integrating whole exome or genome sequencing into routine primary care [12]. In our recent multi-stakeholder paper, we proposed a research-based approach to the return of genome-sequencing results. A genome-first approach can accelerate evaluation of the clinical utility of many genomic applications, including several pharmacogenomic tests and promising genetic risk scores, using randomized trials and implementation science [12].

Third, in the past few years, precision medicine has emerged as a field that allows integration of genomic data with environmental and social information to evaluate personalized prevention and intervention strategies [13]. Similarly, precision public health has emerged as an approach that uses big data to target interventions to the right populations at the right time, to help solve population health concerns and to address health disparities [13]. Applications of pathogen genomics to public health surveillance and outbreak investigations are one example of precision public health. Others include spatial analysis and improved public health surveillance [13].

These topics are only the tip of the iceberg. Clearly, there are now tangible genomic applications that can benefit a larger segment of populations and contribute to the overall mission of public health.

*Muin J. Khoury*

*Section Editor, Genomic epidemiology and public health genomics*

## Pharmacogenomics becomes reality: implementation efforts and personalized approaches

Recently, tremendous progress has been achieved in the implementation of pharmacogenomics (PGx) in clinics. Therapeutic recommendations for more than 40 specific gene–drug pairs have been provided by two consortia, the Clinical Pharmacogenetics Implementation Consortium (CPIC; https://cpicpgx.org/) and the Dutch Pharmacogenetics Working Group (DPWG; https://www.pharmgkb.org/).

The Absorption, Distribution, Metabolism, and Excretion (ADME) of drugs is controlled by approximately 300 genes that have essential consequences for both drug therapy and for the development of new chemical drug entities. Genetic variation in ADME genes contributes significantly to the inter-individual variability of pharmacokinetic and pharmacodynamic processes by producing alternate gene expression patterns and gene functions. Actionable PGx variants are ubiquitous, and four out of five patients are likely to carry a variant with a possible functional effect on ADME targets for commonly prescribed medications [14]. Consequently, a European Consortium, the Ubiquitous Pharmacogenomics (U-PGx) Consortium, is currently implementing pre-emptive PGx testing into routine care for PGx-guided drug therapy within an ongoing multicenter, randomized controlled study [15].

Independent from this activity, there has been a major evolution of PGx research over the past 5 years that is driven by rapidly developing genomic tools. Innovative genotyping arrays and next-generation DNA sequencing (NGS) approaches are used to identify systematically rare variants and gene alterations (e.g. copy number variations) in the human genome that have putative functional consequences for ADME genes [16] and drug response [17]. Although multiple computational methods have been described for and applied in the pharmacogenomic interpretation of NGS data, the elucidation of the functional consequences of PGx data is still challenging [18]. In this context a novel technique, variant abundance by massively parallel sequencing (VAMP-seq), appears to be promising [19]. VAMP-seq, which measures the steady-state abundance of protein variants in cultured human cells, was successfully applied in PGx research recently. As proof of concept, this new technique was used to identify hundreds of unknown missense variants in a highly clinically relevant drug metabolising enzyme, thiopurine S-methyltransferase (TPMT). This approach promises to provide a better understanding of the functional consequences of even cell-type specific single-amino-acid variants in pharmacogenes at scale.

*NUDT15* (*NUDIX HYDROLASE 15*), a genetic variation resulting in no or severely decreased function, is a recent striking PGx discovery [20, 21]. NUDT15 alters thiopurine metabolism, and thiopurines are the mainstay in the treatment of childhood acute lymphoblastic leukaemia and of patients with inflammatory bowel disease. Thiopurine-related hematopoietic toxicity is, however, therapy-limiting and even life-threatening. TPMT is involved in the catabolism of thiopurines, and inherited TPMT deficiency largely explains thiopurine-related myelosuppression in Europeans and Americans [22], whereas the majority of thiopurine intolerance in Asians can be explained by *NUDT15* genetic variants. This justifies the use of pre-emptive *NUDT15*-guided thiopurine, in addition to *TPMT*, to treat malignant and non-malignant disease [23].

Single-analyte biomarkers such as genetic variation are only partially able to explain the heterogeneity in the expression and function of ADME genes. Our understanding of their interaction with non-genetic factors, epigenetic modifications (such as DNA methylation), and metabolic factors is evolving [24]. Multi-analyte signatures derived from complex high-throughput data, which allow patient characterization and prediction of drug response in a more holistic manner, appear to be promising. The strong dependency of personalized drug treatment on computational solutions remains a challenge and modern approaches from data science, specifically multivariate stratification algorithms using techniques such as machine learning or artificial intelligence, are proposed for the prediction of drug response [25]. Prospects at the intersection of machine learning and network biology may have added value in providing a more precise prediction of drug response in the era of personalized medicine [26].

*Matthias Schwab*

*Section Editor, Pharmacogenomics and personalized medicine*

## Metabolomics and proteomics: from the bench to the bedside … almost

The first 10 years of *Genome Medicine* have witnessed huge changes in the availability and complexity of metabolomic and proteomic experiments, which are increasingly used in terms of hypothesis generation or in more targeted approaches in studies of human disease. This progress has been driven by improvements in hardware and software, which have extended the limits of detection and allowed the acquisition of ever larger datasets. Proteomics and metabolomics are now being applied at the epidemiology level. For example, Soranzo and colleagues [27] demonstrated how such data can be used with Mendelian randomization to understand mechanistically how SNPs relate to clinical lipid parameters in cardiovascular disease. The improvements of software have gone hand in hand with the increased availability of data. Although the field has encountered challenges relating to data standardization [28], data sharing is now becoming more commonplace thanks to the combined efforts of funding bodies, researchers, big data initiatives, and publishers. *Genome Medicine* has been at the forefront in ensuring that metabolomic and proteomic datasets are made available for others to data mine. As these datasets become more widely available, we will see them used increasingly to investigate supplementary questions that were not anticipated by those who designed the original experiment.

One of the biggest surprises over the past 10 years has been the importance of the various microbiomes, particularly in the gut microbiome, and their interconnection with host metabolism. This has been particularly true for metabolic diseases such as type 2 diabetes and obesity, but we have also seen the roles of microbiomes in drug metabolism and various cancers. For example, Hale and colleagues [29] combined metabolic modelling and metabolomics to reveal the impact of H_2_S generation on the colon microbiome and in turn how this change interacts with tumour growth. Both metabolomics and proteomics are increasingly being used to map the interactions of humans and the microbes that colonize the various niches of the body, and this work is set to increase as we begin to appreciate how much the microbes inside us play a part in health and disease. The complexity of a host–microbiome network was impressively demonstrated by Grassl and colleagues [30] who pieced together the human and microbiome components of the saliva proteome, including changes that seem to anticipate food consumption. Finally, omics studies are also beginning to map how bacterial and viral infections can hijack host metabolism. Johannes and colleagues [31], using a range of targeted and untargeted metabolomics and lipidomics approaches, demonstrated how hepatitis B infection alters lipid and amino acid metabolism in the liver. This work suggested that a central event in infection is alteration of the glycerol-3-phosphate-NADH shuttle, which in turn affects a variety of lipid pathways including phospholipid ether and triglyceride production.

New applications of metabolomics, lipidomics, and proteomics are being reported continually, but one thing is certain: in the past 10 years, we have only just scratched the surface of what these approaches have to offer.

*Julian L. Griffin*

*Section Editor, Proteomics & metabolomics in medicine*

## Ten years of systems medicine in *Genome Medicine* and beyond

The first issue of *Genome Medicine* in January 2009 coincided with the beginnings of systems medicine, which aims to leverage the advances made in systems biology in the previous decades to deliver innovative solutions for the diagnosis, prognosis, and treatment of human diseases [32]. The field has grown steadily over the past 10 years, empowering the transition from a reactive practice to a proactive practice of medicine, healthcare, and wellbeing, for the benefit of both patients and the healthy population [33].

This transition has been made possible by the combination of advanced biomedical knowledge with a wealth of genomics, transcriptomics, proteomics, metabolomics, and microbiome data. In addition, the constant participatory monitoring of various exposures and lifestyle parameters (such as heart and respiration rate, blood pressure, body temperature and motion) and ambient parameters (through connected wearable devices [34]) has provided the means to disentangle the causative parameters of health maintenance and of disease onset, progression, and response to drug treatments using correlations derived from population-based studies and clinical trials [35]. In the process, multiple challenges have been identified that must be overcome so that we can make sense of big data in health research; there is a need to facilitate open, unencumbered access to personal and population medical data while preserving privacy according to recent pieces of personal data protection legislation [36–38].

In addition, biomedical and computational research infrastructures are leveraging four decades of experience in handling and sharing massive amounts of data in particle physics, which led the Centre Européen de Recherche Nucléaire (CERN) to invent the World Wide Web. This transdisciplinary experience will serve in the development of a health research innovative cloud environment (manuscript submitted). The technological barriers to introducing systems medicine into clinical practice are thus being overcome, as shown by the growing number of successful applications being presented at international conferences [39]. It is likely to take a generation to achieve the training of a new cohort of data scientists, medical doctors, health practitioners, and policy makers who are familiar with ‘advanced intelligence’ and who will work at the crossroads of human wisdom and machine performance, its application to healthcare and wellbeing, and its endorsement by the citizens through a world alliance of health and wellbeing [40].

*Charles Auffray*

*Section Editor, Systems medicine and informatics*

## Abbreviations

ADME: Absorption, Distribution, Metabolism, and Excretion
CNV: Copy number variants
CS: Congenital scoliosis
DD: Developmental delay
ES: Exome sequencing
ID: Intellectual disability
NGS: Next-generation DNA sequencing
*NUDT15*: *NUDIX HYDROLASE 15*
PGx: Pharmacogenomics
TPMT: Thiopurine S-methyltransferase
VAMP-seq: Variant abundance by massively parallel sequencing
